# Efficacy and safety of HT080 for lower urinary tract symptoms associated with benign prostatic hyperplasia: A study protocol for a randomized, double-blind, placebo-controlled trial

**DOI:** 10.1097/MD.0000000000017848

**Published:** 2019-11-11

**Authors:** Jungbin Song, Seung Hwan Lee, Hocheol Kim

**Affiliations:** a Department of Herbal Pharmacology, Kyung Hee University College of Korean Medicine, 26 Kyungheedae-ro, Dongdaemun-gu; b Department of Urology, Yonsei University College of Medicine, 50-1 Yonsei-ro, Seodaemun-gu, Seoul, Republic of Korea.

**Keywords:** benign prostate hyperplasia, *Cinnamomum cassia*, International Prostate Symptom Score, lower urinary tract symptoms, *Rosa Laevigata*

## Abstract

**Background:**

Lower urinary tract symptoms associated with benign prostatic hyperplasia (LUTS/BPH) are one of the most common conditions seen in middle-aged and elderly men and threaten their quality of life. Since conventional pharmacotherapy for LUTS/BPH can lead to sexual side effects, herbal therapies are widely used as complementary and alternative treatments worldwide. HT080 is an herbal extract of *Cinnamomum cassia* and *Rosa Laevigata*, both of which have been used to treat symptoms typically associated with BPH in traditional Asian medicine. The aims of this trial are to assess whether HT080 can alleviate LUTS/BPH in middle-aged and elderly men, and to investigate the safety of HT080.

**Methods/design:**

A double-blind, randomized, placebo-controlled, two-arm parallel group trial will be conducted in men with moderate LUTS/BPH. A total of 100 eligible men aged 40 to 75 years with an International Prostate Symptom Score of 8 to 19 will be randomized in a 1:1 ratio and receive either HT080 (500 mg) or placebo twice a day for 12 weeks. All participants will be evaluated for efficacy and safety at baseline and weeks 6 and 12. The primary endpoint is the change in International Prostate Symptom Score between baseline and week 12. The secondary efficacy variables are uroflowmetry parameters (maximal urinary flow rate and post-void residual volume), serum prostate-specific antigen, testosterone, and dihydrotestosterone levels, the International Index of Erectile Function score, and participant-reported global response assessment scores. The safety assessments include adverse events, laboratory tests results, vital signs, and physical examination.

**Discussion:**

This is a first-in human trial designed to investigate the efficacy and safety of HT080 among middle-aged and elderly men with LUTS/BPH. This prospective study with a double-blind randomized design will provide high-quality evidence supporting the use of HT080 for LUTS/BPH.

**Trial registration:**

Korean Clinical Research Information Service (KCT0004286) Registered September 6, 2019.

## 1 Introduction

Lower urinary tract symptoms (LUTS) are common in middle-aged and elderly men and significantly impair the quality of life.^[1]^ LUTS include storage, voiding, and post-micturition symptoms inducing increased frequency, nocturia, incontinence, weak stream, hesitancy, intermittency, straining to void, terminal dribble, and a sensation of incomplete bladder voiding. LUTS in men generally results from benign prostatic hyperplasia (BPH) which is characterized by hyperproliferation of epithelial and stromal cells of the prostate. The global prevalence of BPH has rapidly increased with aging in the population, thus becoming an economic burden.^[2]^ In Korea, the prevalence of BPH in 2016 increased 2.2-fold that in 2012 and approached 37.5% and 53.8% among men in their 60s and in those above 80 years of age, respectively.^[3]^

Treatment options for LUTS associated with BPH (LUTS/BPH) include watchful waiting, lifestyle modification, medications, and surgery.^[4]^ Two classes of drugs, α-adrenergic receptor blockers and 5α-reductase inhibitors, are used for pharmacotherapy. Unfortunately, these drugs are associated with sexual side effects, including ejaculatory disorders for α-adrenergic receptor blockers and erectile dysfunction, ejaculatory dysfunction, and decreased libido for 5α-reductase inhibitors.^[5]^ This has led a to an increased demand of herbal therapies (*Serenoa repens*, *Pygeum africanum*, *Hypoxis rooperi*, etc.) as complementary and alternative treatments among numerous patients worldwide.^[6,7]^ In Europe and the USA, about half or more of BPH patients use herbal therapies alone or with other drugs.^[6,8]^ In Asian countries including China and Korea, traditional herbal medicine has been used to treat BPH for hundreds of years, and is still commonly used to this day.^[9–11]^

HT080 is an herbal extract of *Cinnamomum cassia* bark and *Rosa laevigata* fruit. It was developed by screening various herbs used in traditional Asian medicine for the treatment of LUTS, with the aim of reducing prostate smooth muscle tone and prostate growth. These two herbs have been traditionally prescribed in combination to treat symptoms typically associated with BPH including frequent urination and nocturia in China.^[12,13]^*C cassia*, commonly called Chinese cassia or cassia cinnamon, is an evergreen tree that originated in southern China and is now cultivated in eastern and southern Asia for its bark, cinnamon. In traditional Korean, Chinese, and Ayurvedic medicine, the bark of *C cassia* has been widely used to treat frequent urination, nocturia, difficulty in urinating, and a weak urine stream in middle-aged and elderly men.^[7,10,14,15]^ A systematic review reported that *C. cassia* is the most frequently used single herb in Chinese herbal medicine for BPH and was used in 17 (54.8%) of 31 studies.^[10]^ Concurrent with these clinical applications, cinnamic acid, the major component of *C cassia*, serves as a potent antagonist of the α_1A_-adrenoceptor in the rat prostate.^[16]^ Furthermore, *C verum*, which is used as a substitute for *C cassia* and vice versa, has reportedly reduced testosterone-induced BPH by downregulating 5α- reductase and androgen receptor expression.^[17]^*R laevigata* is a climbing shrub originating from South China and Southeast Asia, and its fruit has been used to treat frequent urination, urinary incontinence, and sexual dysfunction in traditional Chinese and Korean medicine.^[18,19]^*R Laevigata* reportedly decreases the voiding frequency and increases the inter-micturition interval and urine volume by inhibiting smooth muscle contraction in a mouse pollakiuria model.^[20]^ Furthermore, *R laevigata* has been shown as one of the most potent 5α-reductase inhibitors in a screening assay for anti-5α-reductase, including 162 plants.^[21]^ Although traditional use and preclinical findings support the applications of HT080, a well-designed prospective clinical trial has yet to be conducted to evaluate the efficacy and safety of HT080.

### 1.1 Objectives

This trial primarily aims to determine whether HT080 is superior to the placebo for reducing LUTS, as measured on the basis of the International Prostate Symptom Score (IPSS), in middle-aged and elderly men with moderate LUTS/BPH. The secondary objectives are to investigate the effects of HT080 on uroflowmetry parameters and androgen levels, and to assess the safety of HT080. The exploratory objective is to evaluate whether HT080 affects sexual function.

### 1.2 Design

This study is a double-blind, randomized, placebo-controlled, superiority trial with two parallel groups. Eligible participants will be randomly assigned to either the HT080 or placebo group with a 1:1 allocation ratio and will receive the investigational product for 12 weeks.

## 2 Methods and design

### 2.1 Study setting

Participants will be recruited from Department of Urology, Yonsei University Severance Hospital (Seoul, Republic of Korea). The estimated recruitment period will be 1 year beginning from October 2019. The recruitment will be advertised on hospital bulletin boards and websites. Monthly newsletters will be sent to encourage enrollment until the required sample size is achieved. The first participant has not been enrolled yet.

### 2.2 Eligibility criteria

Following will be the inclusion criteria for the study:

- Men aged 40–75 years
- An IPSS of 8–19
- Prostate size ≥ 20 g
- Availability of written informed consent to participate in the study.

Following will be the exclusion criteria for the study:

- Current treatment for clinically significant acute or chronic cerebrovascular, immune, respiratory, hepatobiliary, kidney and urinary, neurological, musculoskeletal, psychiatric, infectious, or hemato-oncological diseases. (However, inclusion/exclusion from the trial will be at the investigators’ discretion based on participants’ condition).
- Serum prostate-specific antigen level ≥ 10 ng/mL. (However, participants in whom test results are not definitively confirmed as cancer within 6 months prior to study enrollment will be eligible for inclusion in the study).
- Maximal urinary flow rate < 5 mL/s
- Post-void residual urine volume > 200 mL
- Bladder tumors, urinary stones, urethral strictures, bladder neck contracture, inflammation of the lower urinary tract (bladder and urethra), or urinary tuberculosis
- Diagnosis of prostate cancer
- History of genitourinary surgery, including prostate surgery or other invasive procedures for treatment of prostatic conditions (transurethral needle ablation, and laser treatment, among other such therapies)
- Uncontrolled hypertension (systolic blood pressure ≥ 160 mmHg or diastolic blood pressure ≥ 100 mmHg measured after a 10-min rest)
- Uncontrolled diabetes (fasting blood glucose level ≥ 180 mg/dL or administration of drugs for diabetes within the preceding 3 months)
- Thyroid diseases
- Serum creatinine level > 2-fold the upper limit of normal
- Aspartate and alanine aminotransferase levels ≥ 3-fold the upper limit of normal
- Administration of drugs (antimuscarinics and/or others) for benign prostatic hyperplasia (BPH) or intake of functional foods within the preceding 4 weeks
- Participation in other clinical trials within the preceding 3 months or plans to participate in other trials during the present study.
- Unsuitability for study participation based on the investigator's discretion.

### 2.3 Procedures

**Figure 1:**
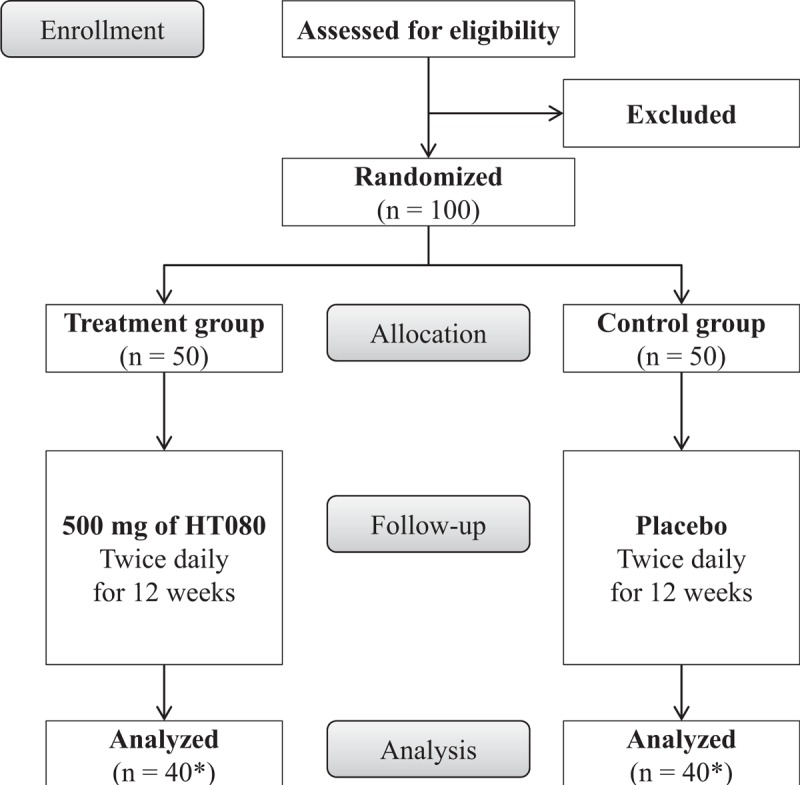
The study flow diagram. ^∗^The number of participants expected to complete this trial.

This trial is designed to provide treatment with either HT080 or placebo in subjects with LUTS/BPH and assess symptom improvement during 12 weeks of treatment. The flow diagram is shown in Figure 1 and the schedule for enrollment, interventions, and assessment of this study is summarized in Table 1. The principle investigator will obtain written informed consent from subjects who voluntarily agreed to participate in this trial before eligibility assessment. After applying inclusion and exclusion criteria, eligible participants will undergo baseline assessments within 14 days of screening visit. Following the administration of HT080 or placebo, participants will visit the hospital at weeks 6 and 12 (visit window ± 7 days) and evaluated for efficacy and safety of the investigational product as shown in Table 1. A 24-hour dietary recall questionnaire will be used on 3 random days during the last week of each time point at baseline and at weeks 6 and 12 to investigate participants’ usual dietary pattern during the trial.

**Table 1:**
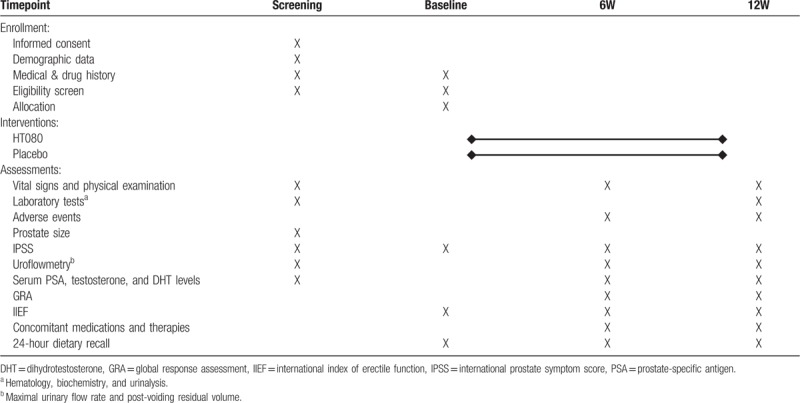
The schedule of enrollment, interventions, and assessments.

### 2.4 Interventions

The investigational product will be provided by COSMAX BIO Inc. (Jecheon, Chungcheongbuk-do, Republic of Korea). The active product is a 500 mg tablet containing 250 mg of HT080 (60 mg of *C cassia* extract and 190 mg of *R laevigata* extract). To ensure blinding, the placebo tablet will be of the same size, shape, and color as the active tablet. Participants will be orally administered 2 active or placebo tablets twice daily (morning and evening before meals) for 12 weeks. HT080 will be administered at a dose of 1000 mg daily. The investigational product will be packaged as a 6-week supply and will be prescribed to participants at baseline and week 6. To improve the adherence to the treatment, participants will be encouraged to adhere to the prescribed dosage regimen, and compliance will be evaluated by counting the number of unused tablets returned at 6 and 12 weeks.

The following treatments will be prohibited during the trial period: drugs such as 5α-reductase inhibitors and α-blockers administered for BPH, drugs administered for nocturnal enuresis or erectile dysfunction, antimuscarinic agents and supplements for prostate health, and procedures such as transurethral needle ablation, prostatectomy, and laser treatment. Intake of functional foods and medications administered before study participation and agents that are not expected to affect the trial results may be permitted at the investigator's discretion. Drugs used for short-term treatment of other diseases will be co-administered after consultation with the investigator. Participants will be instructed to continue their usual dietary pattern and physical activity habits during the trial period and avoid the consumption of products containing *C cassia* and/or *R laevigata*. Concomitant medications and therapies will be monitored at 6 and 12 weeks.

Criteria for discontinuing intervention will include the following:

1. violation of inclusion or exclusion criteria,
2. detection of a systemic disease that had not been identified during participant screening,
3. discontinuation of participation following notice of withdrawal by the participant or his/her legal representative because of unsatisfactory trial results or adverse events during the study period,
4. withdrawal of consent to participate,
5. loss to follow-up,
6. difficulty with administration of the investigational product,
7. intake of other medications, such as 5α-reductase inhibitors, α-blockers, and antimuscarinics that could affect the results of this trial, and (8) trial discontinuation is deemed necessary by the investigator.

### 2.5 Randomization and blinding

Eligible participants will be randomized in a double-blind fashion to HT080 or placebo (1:1 ratio) using a block randomization method. The randomization sequence number will be generated by an independent statistician using the SAS system (SAS Institute Inc., NC). Participants will be assigned a randomization number based on the order of enrollment and will receive the investigational product labeled with the corresponding randomization number during the study period. Block size will not be disclosed to the research personnel for allocation concealment. Packaging of the active product and the placebo will be identical to maintain double-blinding. The randomization sequence will be sealed in opaque envelopes and will not be disclosed until the end of the study, except for cases in which participants develop serious adverse events (medical emergencies). If the investigator concludes that it is necessary to break the code, he/she will notify the contract research organization and the study sponsor regarding premature unbinding, and they will discuss and decide together whether the situation warrants breaking the code. In cases of a code break, the date and reason will be recorded in the case report form, and the corresponding participant will discontinue the study.

### 2.6 Outcome measures

The Korean IPSS questionnaire will be used to assess the primary endpoint. The IPSS, which has been used for decades to assess the severity of LUTS/BPH, contains seven questions on voiding symptoms (incomplete emptying, intermittency, weak stream, and straining) and storage symptoms (frequency, urgency, and nocturia).^[22]^ Each item is scored 0 to 5, giving a maximum total score of 35. A total score of 0 to 7 indicates mild symptoms, 8 to 19 indicates moderate symptoms and 20–35 indicates severe symptoms. Participants will complete the IPSS questionnaire at screening, baseline, and weeks 6 and 12. The primary efficacy endpoint is the change in IPSS at week 12 from the baseline.

As the secondary outcomes, uroflowmetry parameters (maximal urinary flow rate and post-void residual volume) and serum prostate-specific antigen, testosterone, and dihydrotestosterone levels will be measured at screening and weeks 6 and 12. Global response assessment will be completed by participants at weeks 6 and 12 using a five-point scale (markedly worsened, worsened, unchanged, improved, and markedly improved). It has been reported that 70% of BPH patients has an associated erectile dysfunction.^[23]^ Participants will complete the International Index of Erectile Function questionnaire, a multi-dimensional self-report instrument widely used to evaluate male sexual function,^[24,25]^ at baseline and weeks 6 and 12. The safety outcome measures include adverse events, laboratory tests (hematology, biochemistry, and urinalysis) results, vital signs, and physical examination.

### 2.7 Sample size

This trial aims to determine whether HT080 is superior to placebo for reducing the change in IPSSs from baseline to week 12. The sample size has been estimated based on the results of a previous study that had shown an extract of *Ganoderma lucidum* improves IPSSs.^[26]^ Because the standard deviations for changes in IPSSs are not present in this literature, they have been approximated using the confidence intervals and significant probabilities of pre- and post-treatment values for IPSSs. The difference in the mean change in IPSSs between the HT080 and control groups is hypothesized to be 1.18 and the standard deviation is hypothesized to be 1.88. A total of 80 participants, 40 in each group, are required to achieve 80% power with a two-sided significance level of 5%. Considering a dropout rate of 20%, a total of 100 participants (50 in each group) will be enrolled.

### 2.8 Statistical analysis

Efficacy outcomes will be assessed in both full analysis and per-protocol sets. The full analysis set will consist of all randomized participants who are subjected to at least one efficacy evaluation after being administered at least one dose of the study product without failing major inclusion criteria. The per-protocol population will include participants in the full analysis population who complete this trial without major protocol deviations that affect the study results. Safety outcomes will be analyzed in the safety set, which will include all randomized participants who receive at least one dose of study product. Missing values will be handled by the last observation carried forward method.

Continuous variables will be presented with mean, median, standard deviation, and range (min and max), and categorical variables will be presented as frequency and percentage. Continuous variables between the two groups will be compared by the two-sample *t* test or Wilcoxon rank sum test, depending on the normality of distribution, and the differences before and after treatment in each group will be analyzed using a paired t-test or Wilcoxon signed-rank test. Categorical variables will be compared using the chi-square test or Fisher exact test. For primary and secondary efficacy variables, analysis of covariance will be performed to compare the change from baseline to weeks 6 and 12 between the groups while adjusting for baseline values. If necessary, subgroup analysis stratified by baseline characteristics (e.g., age) will be performed. Adverse events will be coded by System Organ Class and Preferred Terms from the Medical Dictionary for Regulatory Activities. Probability values of <.05 with two-tailed tests will be considered significant. The statistical analysis will be performed with SPSS Statistics software version 25 (IBM, Chicago, IL). There will be no interim analyses.

### 2.9 Data management and monitoring

To ensure data quality, regular monitoring by a contract research organization will be conducted in compliance with standard operating procedures until the end of the study. Experienced clinical research associates will verify source documents and case report forms. All documents related to this trial will be kept confidential in a separate facility, and access to the documents will only be allowed for the purposes of trial management. Data quality will be promoted using double data entry and range checks for data values. A data monitoring committee will not be necessary because the study product is low-risk and no interim analyses will be performed. Auditing is not scheduled for this study.

### 2.10 Harms

All adverse events will be recorded in the case report form regardless of causality. The investigator will evaluate the severity of symptoms and determine causal relationship between the study product and the adverse events. If a serious adverse event occurs, the investigator will report to the Institutional Review Board at the earliest and determine whether to continue or stop the trial. Participants will receive financial compensation for injuries found to be due to the study product or procedure.

### 2.11 Withdrawal and dropout

Participants may withdraw from the trial at any time for any reason. The investigator may also withdraw participants from the trial due to medical or administrative reasons (adverse events, continuous protocol violations, etc.). Reasons for withdrawal and dropout will be recorded in the case report form.

### 2.12 Ethics and dissemination

This protocol (ver. 1.0, issue date 11 Jan 2019) has been approved by the Institutional Review Board of Yonsei University Severance Hospital (approval no. 4-2019-0115) and is registered with Clinical Research Information Service (Identifier: KCT0004286). The trial will be conducted in accordance with the *Declaration of Helsinki* and the Korean Good Clinical Practice guidelines. All participants will be provided detailed information about the trial and the principle investigator will obtain handwritten informed consent from each participant before proceeding with the trial. To protect participants’ identity, subject identification codes will be used instead of participants’ names in all documents. The sponsor and principal investigator will have access to the final dataset. The full protocol and datasets will not be publicized but available from the corresponding author on reasonable request. A model consent form is also available from the corresponding author. Results from this study will be presented at an academic conference or published in a peer-reviewed journal.

## 3 Discussion

BPH is a common disease affecting the overall quality of life in industrial countries. Although traditional herbal medicines are widely used to treat LUTS/BPH in Asian countries including Korea, their efficacy remains unclear owing to the poor quality of clinical trials.^[10]^ There is an unmet need to investigate the efficacy and safety of herbal medicines in well-designed and rigorous studies. Herein, we designed the study protocol of the first double-blind, randomized, placebo-controlled trial of HT080 as a single intervention for Korean men with LUTS/BPH.

One of the limitations of this trial is that the treatment duration is 3 months, thus providing short-term outcomes. Furthermore, this study has a relatively small sample size, albeit yielding a statistical power of 80%. The present results will warrant confirmation with further long-term studies with larger sample sizes. Nevertheless, this study has several strengths; it is a prospective study with a double-blind randomized design. In addition, the IPSS questionnaire, the primary efficacy measure of this trial, has been established worldwide as a useful tool to determine the extent of LUTS/BPH, and the validity and reliability of its Korean version has been confirmed.^[22,27]^ We intend to use not only subjective measures (such as IPSS, global response assessment) but also objective measures, such as maximal urinary flow rate and post-void residual volume, to yield outcomes on voiding dysfunction. In addition, our inclusion criterion of an IPSS of 8–19 reflects men with moderate symptoms commonly observed in clinical practice.

In conclusion, this is a first-in-human trial of HT080 in men with LUTS/BPH. The results of this trial will provide high-quality evidence of therapeutic benefit of HT080 for the treatment of LUTS/BPH.

## Acknowledgments

We would like to thank all the participants and research personnel for their support of this study.

## Author contributions

**Conceptualization:** Hocheol Kim

**Methodology:** Jungbin Song, Seung Hwan Lee

**Project administration:** Seung Hwan Lee

**Supervision:** Seung Hwan Lee

**Writing – original draft:** Jungbin Song

**Writing – review & editing:** Seung Hwan Lee, Hocheol Kim

Hocheol Kim orcid: 0000-0002-6690-0226.
